# Predictors of hypotension during anesthesia induction in patients with hypertension on medication: a retrospective observational study

**DOI:** 10.1186/s12871-022-01899-9

**Published:** 2022-11-11

**Authors:** Takayuki Hojo, Yukifumi Kimura, Makiko Shibuya, Toshiaki Fujisawa

**Affiliations:** Department of Dental Anesthesiology, Faculty of Dental Medicine and Graduate School of Dental Medicine, Hokkaido University, Nishi 7, Kita 13, Kita-Ku, Sapporo, Japan

**Keywords:** Anesthesia Induction, General anesthesia, Hypotension, Antihypertensive medication, Predictor, Half-life

## Abstract

**Background:**

Hypotension during anesthesia induction is a common event, and occurs more frequently in patients with hypertension than in healthy individuals. Intraoperative hypotension in non-cardiac surgery is reportedly associated with various postoperative complications. However, the predictors of hypotension during anesthesia induction in patients with hypertension have not yet been ascertained. Therefore, we aimed to determine the predictors of hypotension during anesthesia induction in patients with hypertension on medication focusing on the half-life of the medication used.

**Methods:**

In this retrospective observational study, we enrolled patients with hypertension on medication who underwent general anesthesia for oral and maxillofacial surgery between January 1, 2013, and December 31, 2019. Multivariable logistic regression analysis was conducted to test for associations between clinical factors and hypotension during anesthesia induction in patients with hypertension on medication.

**Results:**

A total of 395 patients were included in this study. The risk factors for hypotension during anesthesia induction in patients with hypertension on medication were pre-induction mean arterial blood pressure (adjusted unit odds ratio, 0.96 [95% confidence interval, 0.94 to 0.98]), female sex (adjusted odds ratio [aOR], 1.63 [1.03 to 2.57]), regular use of angiotensin receptor blockers (ARBs)/angiotensin-converting enzyme inhibitors (ACE-Is) with a long half-life (vs. no regular use of ARBs/ACE-Is aOR, 4.02 [1.77 to 9.12]; vs. regular use of ARBs/ACE-Is with a short-to-middle half-life aOR, 3.17 [1.46 to 6.85]), and regular use of beta blockers (aOR, 2.45 [1.19 to 5.04]). Regular use of calcium channel blockers (aOR, 0.44 [0.25 to 0.77]) was a suppressive factor for hypotension during anesthesia induction in patients with hypertension.

**Conclusions:**

In patients with hypertension on medication, regular use of ARBs/ACE-Is with a long half-life, regular use of beta blockers, low pre-induction mean arterial blood pressure, and female sex were risk factors for hypotension during anesthesia induction. Notably, regular use of ARBs/ACE-Is with a long half-life was a high-risk factor for hypotension during anesthesia induction in patients with hypertension on medication even after a 24-h preoperative withdrawal period.

**Supplementary Information:**

The online version contains supplementary material available at 10.1186/s12871-022-01899-9.

## Background

Hypotension often occurs during anesthesia induction in all adult patients, with an incidence of approximately 20% [1–3]. It is reported that a substantial fraction of intraoperative hypotension in noncardiac surgery occurs before the start of surgery [4]. Intraoperative hypotension in noncardiac surgery has been reported to be associated with various postoperative complications such as mortality [5], myocardial damage [5, 6], and acute kidney injury [5–9]. Recently, there have been an increasing number of studies on predictors of hypotension during anesthesia induction [1–3, 10, 11].

Hypotension during anesthesia induction has been reported to occur frequently in patients with hypertension [12, 13]. Intraoperative hypotension has been known to increase the risk of organ damage in patients with hypertension [14]. Angiotensin receptor blockers (ARBs) and angiotensin-converting enzyme inhibitors (ACE-Is) are often used to treat hypertension. The risk of intraoperative hypotension associated with the preoperative continuation of these drugs is still under investigation [15–17]. We previously reported a case wherein the regular use of ARBs with a long half-life might have caused refractory hypotension during anesthesia induction, even when ARBs had been withdrawn 24 h prior to general anesthesia induction [18]. However, a multivariable analysis of predictors of hypotension during anesthesia induction in patients with hypertension on medication has not yet been conducted.

We hypothesized that there are unknown predictors of hypotension during anesthesia induction in patients with hypertension on medication. We aimed to determine the predictors of hypotension during anesthesia induction in patients with hypertension on medication such as ARBs and ACE-Is, focusing on the half-life of the medication used.

## Methods

### Study design and ethical considerations

The protocol of this retrospective observational study was approved by the local ethics committee (Institutional Review Board of Hokkaido University Hospital; clinical study code, 021–0038), and the requirement for written informed consent was waived by the Institutional Review Board. We publicly announced this study on the homepage of the website of our institute and announced that participants could opt out of it. None of the subjects opted out of participating in this study. This study was conducted in accordance with the principles of the Helsinki Declaration and reported in accordance with the STROBE Statement. The data used for analysis were obtained from digitized anesthesia records and digitized medical records.

### Inclusion and exclusion criteria

A total of 428 patients with hypertension who were on medication and had undergone general anesthesia for oral and maxillofacial surgery between January 1, 2013, and December 31, 2019, were screened against the eligibility criteria. Patients with hypertension on medication were defined as those who had been diagnosed by physicians and were receiving medication. The exclusion criteria were as follows: awake fiberoptic intubation for a difficult airway, anesthesia induction with midazolam or thiamylal, age < 20 years, missing data, and patients not on regular medication (ARBs, ACE-Is, calcium channel blockers [CCBs], beta blockers, and/or diuretics).

### Definition of arterial hypotension during anesthesia induction

A substantial fraction of intraoperative hypotension in noncardiac surgery reportedly occurs before the start of surgery [4]. A recent study found that absolute hypotension was more strongly associated with clinical prognosis compared with relative hypotension [7]. Therefore, we used the cutoff value for absolute hypotension as the definition of hypotension during anesthesia induction in this study. Since this study focused on anesthesia induction, which is a short period during general anesthesia, the definition of hypotension during anesthesia induction used in this study was based on previous studies that reported the risk of postoperative complications related to brief episodes of intraoperative hypotension. We mainly referred to three articles: a systematic review of the increased risk of mortality, acute kidney injury, and myocardial injury when an intraoperative mean arterial blood pressure (MAP) of < 55 mmHg is sustained for > 5 min [5]; a study reporting that an intraoperative MAP < 55 mmHg sustained for > 10 min increases the risk of acute kidney injury [9]; and a study reporting that an intraoperative MAP of < 55 mmHg sustained for just 1–5 min increases the risk of acute kidney and myocardial injury [6]. Furthermore, we considered that even the shortest durations of hypotension mentioned in these articles may increase the risk of postoperative complications such as acute kidney injury and myocardial damage in patients with hypertension on medication. Therefore, hypotension during anesthesia induction was defined as MAP values of < 55 mmHg for > 1 min within 30 min of propofol administration for anesthesia induction (before the start of surgery). Arterial blood pressure was measured using oscillometric blood pressure cuffs at regular intervals of 2.5 min and at any other time-points when the anesthesiologist deemed it necessary. Arterial blood pressure was automatically recorded every minute in digitized anesthesia records. Vasopressors were administered at the discretion of the anesthesiologist.

### Study outcomes

The primary outcome of this study was to identify predictors of hypotension during anesthesia induction in patients with hypertension on medication within 30 minutes of propofol administration (before the start of surgery) by multivariable logistic regression analysis. The secondary outcome of this study was to identify differences in patient background and clinical factors between patients with and without hypotension (hypotension group vs. non-hypotension group) during anesthesia induction.

### Evaluation methods

The objective variable was whether the MAP decreased to 55 mmHg or lower for > 1 minute within 30 minutes of propofol administration (before the start of surgery). The explanatory variables, which were selected based on previous reports [1–3, 18, 19], were as follows: age; sex; body mass index; American Society of Anesthesiologists physical status; diabetes mellitus; revised cardiac risk index [20] score > 0; ARBs/ACE-Is use, classified as no regular use, short-to-middle half-life, and long half-life (Table 1); CCB use; diuretic use; beta blocker use; pre-induction MAP; propofol dose; fentanyl dose; and remifentanil dose. While ARBs and ACE-Is were withheld for 24 hours prior to general anesthesia induction, CCBs, diuretics, and beta blockers were not. Hydration was stopped and fasting was initiated 3 and 8 hours before general anesthesia, respectively. No anxiolytics were administered before general anesthesia.

**Table 1:** Half-lives of the ARBs/ACE-Is

Groups	ARBs / ACE-Is	t_1/2_ (h)
Short-to-middle half-life group	Losartan	2
	Quinapril	2 (Quinaprilat)
	Aracepril	4 (Captopril)
	Valsartan	6
	Imidapril	8 (Imidaprilat)
	Candesartan	9
	Enalapril	11 (Enalaprilat)
	Olmesartan	13
	Azilsartan	13
	Irbesartan	11–15
Long half-life group	Telmisartan	24
	Cilazapril	44 (Cilazaprilat)

The name of the active metabolite is mentioned in parentheses

*ARB* Angiotensin receptor blocker, *ACE-I*, Angiotensin-converting enzyme inhibitor

### Statistical analysis

Descriptive data are presented as medians with 25^th^–75^th^ percentile ranges for continuous data and as frequencies with percentages for categorical data. Differences in patient background and clinical factors between patients with and without hypotension (hypotension group vs. non-hypotension group) during anesthesia induction were evaluated using the Mann–Whitney U test for continuous variables and Fisher’s exact test for categorical variables. Multivariable logistic regression analysis was conducted to identify factors significantly associated with hypotension during anesthesia induction in patients with hypertension. The odds ratios were adjusted for all explanatory variables in the multivariable logistic regression model. As effect measures, we present adjusted odds ratios (aORs) and 95% confidence intervals (CIs). Calculations were performed using JMP^TM^ Pro14 (SAS Institute Inc., Cary, NC, USA). Statistical significance was set at *P* < 0.05.

## Results

**Fig. 1:**
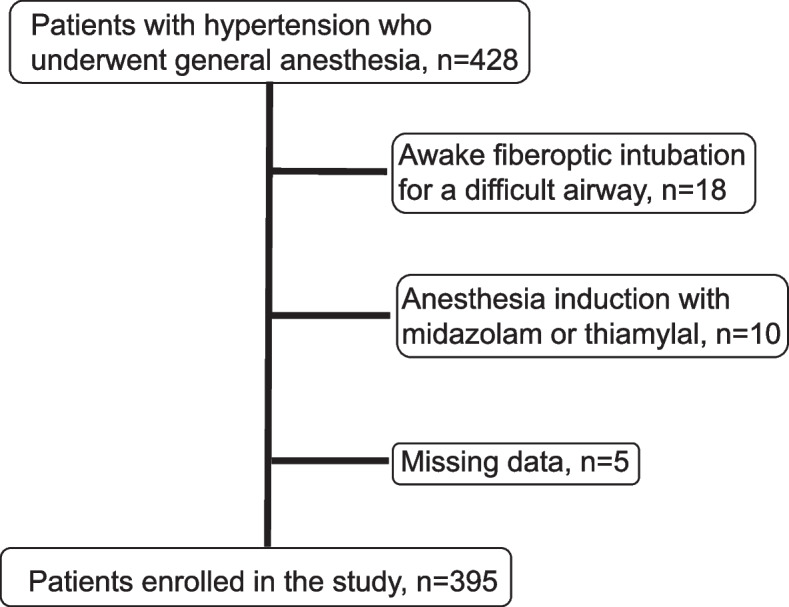
Patient enrollment flow chart

The patient selection flow chart is shown in Fig. 1. After excluding 18 patients who underwent awake fiberoptic intubation for a difficult airway, 10 patients in whom midazolam or thiamylal was used for anesthesia induction, and 5 patients with incomplete data, we enrolled 395 patients in this study.

Hypotension during anesthesia induction was observed in 47.6% (*n* = 188) of the patients. The participants’ baseline characteristics and comparisons between the hypotension and non-hypotension groups are shown in Table 2. Univariate analysis indicated that age and the proportions of females and patients with hyperlipidemia, chronic kidney disease, regular use of ARBs/ACE-Is with a long half-life, regular use of beta blockers, and multiple therapies were significantly higher in patients with hypertension who experienced hypotension during anesthesia induction compared with patients who did not. Details of hypotension during anesthesia induction are shown in Table 3. The median length of time during which the MAP was < 55 mmHg was 4 (25^th^–75^th^ percentiles, 2–8) minutes in the hypotension group.

**Table 2:** Patients’ baseline and anesthesia-related characteristics

Items	All *n* = 395	Hypotension *n* = 188	Non-hypotension *n* = 207	*P*-value
Age (years)	70 [61–78]	73 [63–80]	69 [60–76]	0.017
Female sex	184 (46.6)	101 (53.7)	83 (40.1)	0.009
BMI (kg/m^2^)	24.5 [22.2–27.0]	24.7 [22.2–27.0]	24.5 [22.0–26.7]	0.540
ASA PS III (vs. II)	10 (2.5)	7 (3.7)	3 (1.4)	0.203
DM	101 (25.6)	53 (28.2)	48 (23.2)	0.299
RCRI > 0	99 (25.1)	52 (27.7)	47 (22.7)	0.296
Hyperlipidemia	89 (22.5)	56 (29.8)	33 (15.9)	0.001
CKD (eGFR < 60 mL/min/1.73 m^2^)	118 (29.9)	66 (35.1)	52 (25.1)	0.036
Heart failure	2 (0.5)	2 (1.1)	0 (0)	0.226
Ischemic heart disease	41 (10.4)	23 (12.2)	18 (8.7)	0.322
Cerebrovascular disease	52 (13.2)	24 (12.8)	28 (13.5)	0.882
Long-term medication use				
ARBs/ACE-Is				
No regular use	135 (34.2)	50 (26.6)	85 (41.1)	0.003
Short-to-middle half-life	216 (54.7)	107 (56.9)	109 (52.7)	0.419
Long half-life	44 (11.1)	31 (16.5)	13 (6.3)	0.001
CCBs	299 (75.7)	128 (68.1)	171 (82.6)	0.001
Diuretics	61 (15.4)	29 (15.4)	32 (15.5)	1.000
Beta blockers	47 (11.9)	32 (17.0)	15 (7.2)	0.003
Monotherapy	181 (45.8)	75 (39.9)	106 (51.2)	0.026
Pre-induction SAP (mmHg)	145 [132–159]	142 [128–158]	147 [133–160]	0.009
Pre-induction MAP (mmHg)	92 [86–102]	89 [82–97]	96 [89–106]	< 0.001
Pre-induction DAP (mmHg)	78 [69–86]	74 [65–81]	82 [72–88]	< 0.001
Baseline SAP (mmHg)	137 [124–148]	134 [120–145]	140 [129–151]	< 0.001
Baseline DAP (mmHg)	80 [71–89]	76 [67–86]	83 [74–91]	< 0.001
Propofol (mg/kg)	1.51 [1.25–1.79]	1.47 [1.21–1.74]	1.54 [1.34–1.84]	0.006
Fentanyl (µg/kg)	1.56 [1.37–1.86]	1.54 [1.26–1.87]	1.58 [1.41–1.86]	0.069
Remifentanil (µg/kg)	3.1 [2.6–3.7]	3.0 [2.4–3.5]	3.2 [2.7–3.8]	0.011
Sevoflurane (vs Desflurane)	239 (60.5)	110 (58.5)	129 (62.3)	0.471

Data are presented as medians [25^th^–75^th^ percentile] or numbers (percentages). For categorical data, Fisher’s exact test was used. For continuous data, the Mann–Whitney U-test was used

*BMI* Body mass index, *ASA PS* American Society of Anesthesiologists physical status, *DM* Diabetes mellitus, *RCRI* Revised cardiac risk index, *CKD* Chronic kidney disease, *eGFR* Estimated glomerular filtration rate, *ARB* Angiotensin receptor blocker, *ACE-I* Angiotensin-converting enzyme inhibitor, *CCB* Calcium channel blocker, *SAP* Systolic arterial blood pressure, *MAP* Mean arterial blood pressure, *DAP* Diastolic arterial blood pressure

**Table 3:** Details of hypotension during anesthesia induction

Items	Hypotension *n* = 188	Non-hypotension *n* = 207
Minimum MAP (mmHg)	49 [46–52]	60 [57–65]
Length of time with MAP < 55 mmHg (min)	4 [2–8]	0

Data are presented as medians [25^th^–75^th^ percentile]

*MAP* Mean arterial blood pressure

Multivariable logistic regression analysis revealed that the factors significantly associated with hypotension during anesthesia induction in patients with hypertension on medication were pre-induction MAP (adjusted unit odds ratio, 0.96 [95% CI, 0.94 to 0.98]), female sex (aOR, 1.63 [1.03 to 2.57]), regular use of ARBs/ACE-Is with a long half-life (vs. no regular use of ARBs/ACE-Is aOR, 4.02 [1.77 to 9.12]; vs. regular use of ARBs/ACE-Is with a short-to-middle half-life aOR, 3.17 [1.46 to 6.85]), regular use of CCBs (aOR, 0.44 [0.25 to 0.77]), and regular use of beta blockers (aOR, 2.45 [1.19 to 5.04]) (Table 4) (see Supplementary Table 1 in Additional file 1). None of variance inflation factors exceeded 3, suggesting that there was no multicollinearity among the explanatory variables.

**Table 4:** Explanatory variables and variable categories significantly associated with hypotension during anesthesia induction in patients with hypertension

Explanatory variables	aOR (95% CI)	*P*-value
**Pre-induction MAP (mmHg)**	0.96 (0.94 to 0.98)	< 0.001
**Female sex**	1.63 (1.03 to 2.57)	0.037
**Use of ARBs/ACE-Is with a long half-life**		
vs. no regular use of ARBs/ACE-Is	4.02 (1.77 to 9.12)	< 0.001
vs. use of ARBs/ACE-Is with a short-to-middle half-life	3.17 (1.46 to 6.85)	0.003
**Use of CCBs**	0.44 (0.25 to 0.77)	0.004
**Use of beta blockers**	2.45 (1.19 to 5.04)	0.015

The odds ratios were adjusted for all explanatory variables in the multivariable logistic regression model

The explanatory variables were as follows: age; sex; body mass index; American Society of Anesthesiologists physical status; diabetes mellitus; revised cardiac risk index score > 0; ARBs/ACE-Is use, classified as no regular use, short-to-middle half-life, and long half-life; CCB use; diuretic use; beta blocker use; pre-induction MAP; propofol dose; fentanyl dose; and remifentanil dose

*aOR *Adjusted odds ratio, *CI *Confidence interval, *MAP *Mean arterial blood pressure, *ARB *Angiotensin receptor blocker, *ACE-I *Angiotensin-converting enzyme inhibitor, *CCB *Calcium channel blocker

Details of the use of vasopressors during anesthesia induction are shown in Supplementary Table 2 (see Additional file 2). Details regarding monotherapy and multiple therapies are shown in Supplementary Tables 3 and 4, respectively (see Additional files 3 and 4, respectively). Furthermore, baseline blood pressure data of patients receiving monotherapy and multiple therapies are shown in Supplementary Table 5 (see Additional file 5).

## Discussion

We found that regular use of ARBs/ACE-Is with a long half-life, regular use of beta blockers, low pre-induction MAP, and female sex are risk factors and regular use of CCBs is a suppressive factor for hypotension during anesthesia induction in patients with hypertension on medication.

Considering our finding that regular use of ARBs/ACE-Is with a long half-life increases the risk of hypotension during anesthesia induction in patients with hypertension on medication, it is necessary to consider the pros and cons of preoperative ARBs/ACE-Is withdrawal and the half-lives of ARBs/ACE-Is. First, although it has been reported that withdrawal of ARBs/ACE-Is for 24 h before surgery is effective for avoiding intraoperative hypotension, it has further been reported that ARBs/ACE-Is withdrawal is not necessary, and no consensus has yet been reached [15–17]. In a recent report that preoperative withdrawal of ARBs/ACE-Is is not required to avoid intraoperative hypotension, it was suggested that individual perioperative risk factors should be carefully considered when deciding whether to continue or withhold ARBs/ACE-Is before surgery [17]. In this study, we included a 24-h washout period before general anesthesia for patients who regularly used ARBs/ACE-Is. Second, although the half-life of ARBs/ACE-Is varies from 2 to 44 h [21–26] as shown in Table 1, previous reports have used a uniform washout period. We previously reported a case wherein the regular use of ARBs with a long half-life might have caused refractory hypotension during anesthesia induction, even when ARBs were withheld for 24 h prior to general anesthesia induction [18]. Telmisartan has the longest half-life among all the currently used ARBs, and it accumulates in the plasma with regular use, which prolongs its half-life [26]. Therefore, with a half-life of 24 h set as a cutoff value, we divided the regular use of ARBs/ACE-Is into long half-life and short-to-middle half-life. We found that patients receiving ARBs/ACE-Is with a long half-life had greater odds (aOR, 3.17) of experiencing hypotension during anesthesia induction than those receiving ARBs/ACE-Is with a short-to-middle half-life. Therefore, the washout period of ARBs/ACE-Is, especially those with a long half-life of 24 h or more, should be carefully adjusted by the attending physician to avoid hypotension during anesthesia induction.

Continuation of beta blocker use in the preoperative period has been found to reduce the risk of postoperative cardiovascular complications [27]. Therefore, in accordance with the American College of Cardiology/American Heart Association guidelines [27], all patients who regularly used beta blockers in this study continued to receive them in the preoperative period. Consequently, regular use of beta blockers was found to predict hypotension during anesthesia induction in patients with hypertension on medication. A previous report also found that perioperative administration of beta blockers caused intraoperative hypotension [28]. A recent study has recommended that decisions regarding the preoperative withdrawal or continuation of beta blockers should be made based on the revised cardiac risk index score and that these decisions should be made in consultation with the attending physician [29]. When the administration of beta blockers is continued preoperatively, it is necessary to monitor the patient for hypotension during anesthesia induction.

We found that regular use of CCBs may reduce the risk of hypotension during anesthesia induction in patients with hypertension on medication. The Japanese Society of Hypertension guidelines for the management of hypertension recommends CCBs, ARBs/ACE-Is, or low-dose diuretics as the first-line drugs in the treatment of hypertension in patients without compelling indications [30]. If the antihypertensive effect is insufficient, concomitant use of a different class of antihypertensive drug at a low dose is recommended [30]. The most commonly used drugs by patients receiving monotherapy in this study were CCBs. Patients receiving monotherapy as drug therapy for hypertension reportedly have a lower risk of intraoperative hypotension than that of patients receiving multiple therapies [31, 32]. However, since we did not include whether patients were receiving monotherapy or multiple therapies as an explanatory variable in the multivariate logistic regression analysis of this study, it is difficult to demonstrate how the large proportion of CCBs users among patients receiving monotherapy affected our finding that regular use of CCBs may be a suppressive factor of hypotension during anesthesia induction. All the CCBs used by the patients in this study were dihydropyridines. Previous studies, including a meta-analysis of perioperative CCB use, have found no relationship between regular use of CCBs and hypotension during anesthesia induction [1–3, 11, 19, 33]. However, this result might have been due to the use of diltiazem, and the relationship between intraoperative hypotension and dihydropyridines, which are widely used to treat hypertension, is largely unknown [27, 33]. It is difficult to explain why regular use of dihydropyridines may be a suppressive factor for hypotension during anesthesia induction in patients with hypertension on medication based on the results of this study alone. Future studies of the relationship between dihydropyridines and intraoperative hypotension are required.

We found that a low pre-induction MAP increased the risk of hypotension during anesthesia induction in patients with hypertension on medication. This is consistent with the findings of previous studies, although these studies also included patients other than those with hypertension [1, 2, 11]. Therefore, it may be inferred that a low pre-induction blood pressure increases the risk of hypotension during anesthesia induction, regardless of whether the patients are being treated for hypertension.

Our findings suggested that female sex is a risk factor for hypotension during anesthesia induction in patients with hypertension on medication. However, it is difficult to explain this finding solely based on the results of this study.

Age has been reported as a predictor of hypotension during anesthesia induction in previous studies, although these studies also included patients other than those with hypertension [1–3, 11, 19]. However, in this study, age was not a predictor of hypotension during anesthesia induction in patients with hypertension on medication. Reich et al. [2] have shown that the risk factor for hypotension during anesthesia induction is an age of ≥ 50 years. The overall age range of the patients in this study was higher than that in previous reports because our study was limited to patients with hypertension on medication. In this study, 38 patients, < 10% of the total study population, were < 50 years old. Therefore, it is possible that age was not a predictor of hypotension during anesthesia induction in this study.

In this study, no dose of propofol, fentanyl, or remifentanil was a predictor of hypotension during anesthesia induction in patients with hypertension on medication. Since the participants in this study were patients with hypertension on medication, each anesthesiologist titrated the dose of each anesthetic to avoid hypotension during anesthesia induction, which may have influenced the results.

This study had several limitations. First, the majority of anesthesiologists would have immediately administered vasopressors when the patient’s systolic blood pressure dropped below 90 mmHg in this study. However, there were no common criteria for the administration of vasopressors, which was done at the discretion of each anesthesiologist in this study. There might have been patients who had been treated with vasopressors before their MAP decreased below 55 mmHg. Therefore, it is possible that the number of patients with hypotension during anesthesia induction was much higher. Second, this was a single-center study, and we only included patients undergoing oral and maxillofacial surgery. Although it would be ideal to study all noncardiac surgeries, including oral and maxillofacial surgery, we could only retrieve data from patients undergoing dental anesthesia at our institution. Further multi-center studies will validate our findings.

## Conclusions

This study revealed that regular use of ARBs/ACE-Is with a long half-life, regular use of beta blockers, low pre-induction MAP, and female sex were risk factors for hypotension during anesthesia induction in patients with hypertension on medication. Notably, regular use of ARBs/ACE-Is with a long half-life was a high-risk factor for hypotension during anesthesia induction in patients with hypertension on medication even after a 24-h preoperative withdrawal period.

## Supplementary Information

**Additional file 1:** **Supplementary Table 1.** Explanatory variables and variable categories not associated with hypotension during anesthesia induction in patients with hypertension.

**Addtional file 2:** **Supplementary Table 2.** Details of the use of vasopressors during anesthesia induction.

**Addtional file 3:** **Supplementary Table 3. **Details of monotherapy.

**Additional file 4:** **Supplementary Table 4.** Details of multiple therapies.

**Additional file 5:** **Supplementary Table 5.** Baseline blood pressure data of patients receiving monotherapy and multiple therapies.

## Data Availability

The datasets used and analyzed during the current study are available from the corresponding author on reasonable request.

## Abbreviations

ACE-I: Angiotensin-converting enzyme inhibitor
aOR: Adjusted odds ratio
ARB: Angiotensin receptor blocker
CCB: Calcium channel blocker
CI: Confidence interval
MAP: Mean arterial blood pressure
