# Supplementary material for: Predictors of hypotension during anesthesia induction in patients with hypertension on medication: a retrospective observational study
Source: BMC Anesthesiol. 2022 Nov 11;22:343. doi: 10.1186/s12871-022-01899-9 (PMC9650866; doi:10.1186/s12871-022-01899-9)
Supplement: Supplementary file 1 — Additional file 1: Supplementary Table 1. Explanatory variables and variable categories not associated with hypotension during anesthesia induction in patients with hypertension. [file 12871_2022_1899_MOESM1_ESM.docx]

Supplementary Table 1 Explanatory variables and variable categories not associated with hypotension during anesthesia induction in patients with hypertension

| Explanatory variables | aOR (95% CI) | P-value |
| --- | --- | --- |
| **Age** | 1.00 (0.97 to 1.02) | 0.762 |
| **BMI** | 0.99 (0.92 to 1.06) | 0.713 |
| **ASA PS Ⅲ (vs Ⅱ)** | 2.13 (0.45 to 10.07) | 0.341 |
| **DM** | 1.15 (0.69 to 1.91) | 0.598 |
| **RCRI score >0** | 0.87 (0.50 to 1.52) | 0.614 |
| **Use of ARBs/ACE-Is with a short-to-middle half-life** |  |  |
| vs. no regular use of ARBs/ACE-Is | 1.27 (0.76 to 2.12) | 0.363 |
| **Diuretics** | 0.62 (0.32 to 1.18) | 0.143 |
| **Propofol (mg/kg)** | 0.56 (0.22 to 1.46) | 0.238 |
| **Fentanyl (µg/kg)** | 0.67 (0.42 to 1.06) | 0.086 |
| **Remifentanil (µg/kg)** | 0.99 (0.81 to 1.21) | 0.916 |

The odds ratios were adjusted for all explanatory variables in the multivariable logistic regression model.

The explanatory variables were as follows: age; sex; BMI; ASA PS; DM; RCRI score >0; ARBs/ACE-Is use, classified as no regular use, short-to-middle half-life, and long half-life; calcium channel blocker use; diuretic use; beta blocker use; pre-induction mean arterial blood pressure; propofol dose; fentanyl dose; and remifentanil dose.

aOR, adjusted odds ratio; CI, confidence interval; BMI, body mass index; ASA PS, American Society of Anesthesiologists physical status; DM, diabetes mellitus; RCRI, revised cardiac risk index; ARB, angiotensin receptor blocker; ACE-I, angiotensin-converting enzyme inhibitor
