# Supplementary material for: Predictors of hypotension during anesthesia induction in patients with hypertension on medication: a retrospective observational study
Source: BMC Anesthesiol. 2022 Nov 11;22:343. doi: 10.1186/s12871-022-01899-9 (PMC9650866; doi:10.1186/s12871-022-01899-9)
Supplement: Supplementary file 2 — Addtional file 2: Supplementary Table 2. Details of the use of vasopressors during anesthesia induction. [file 12871_2022_1899_MOESM2_ESM.docx]

Supplementary Table 2 Details of the use of vasopressors during anesthesia induction

| Items | Hypotension n=188 | Non-hypotension n=207 |
| --- | --- | --- |
| Use of vasopressors |  |  |
| Ephedrine | 170 (90.4) | 120 (58.0) |
| Ephedrine total dose (mg) | 12 [8–16] | 8 [4–8] |
| Phenylephrine | 49 (26.1) | 13 (6.3) |
| Phenylephrine total dose (µg) | 200 [100–300] | 50 [50–150] |
| Dopamine | 2 (1.1) | 0 (0) |
| Dobutamine | 0 (0) | 0 (0) |
| Noradrenaline | 0 (0) | 0 (0) |

Data are presented as medians [25^th^–75^th^ percentile] or numbers (percentages).
