# Supplementary material for: Predictors of hypotension during anesthesia induction in patients with hypertension on medication: a retrospective observational study
Source: BMC Anesthesiol. 2022 Nov 11;22:343. doi: 10.1186/s12871-022-01899-9 (PMC9650866; doi:10.1186/s12871-022-01899-9)
Supplement: Supplementary file 3 — Addtional file 3: Supplementary Table 3. Details of monotherapy. [file 12871_2022_1899_MOESM3_ESM.docx]

Supplemental Table 3 Details of monotherapy

| Items | Hypotension n=75 | Non-hypotension n=106 |
| --- | --- | --- |
| ARBs/ACE-Is |  |  |
| Short-to-middle half-life | 29 | 26 |
| Long half-life | 7 | 4 |
| CCBs | 36 | 73 |
| Diuretics | 0 | 2 |
| Beta blockers | 3 | 1 |

ARB, angiotensin receptor blocker; ACE-I, angiotensin-converting enzyme inhibitor; CCB, calcium channel blocker
