# Supplementary material for: Predictors of hypotension during anesthesia induction in patients with hypertension on medication: a retrospective observational study
Source: BMC Anesthesiol. 2022 Nov 11;22:343. doi: 10.1186/s12871-022-01899-9 (PMC9650866; doi:10.1186/s12871-022-01899-9)
Supplement: Supplementary file 4 — Additional file 4: Supplementary Table 4. Details of multiple therapies. [file 12871_2022_1899_MOESM4_ESM.docx]

Supplemental Table 4 Details of multiple therapies

| Items | Hypotension n=113 | Non-hypotension n=101 |
| --- | --- | --- |
| ARBs/ACE-Is with a short-to-middle half-life   + Beta blockers | 5 | 0 |
| ARBs/ACE-Is with a shot-to-middle half-life  + Beta blockers + CCBs | 6 | 8 |
| ARBs/ACE-Is with a shot-to-middle half-life  + Beta blockers + CCBs + Diuretics | 3 | 2 |
| ARBs/ACE-Is with a shot-to-middle half-life  + Beta blockers + Diuretics | 2 | 0 |
| ARBs/ACE-Is with a shot-to-middle half-life  + CCBs | 48 | 54 |
| ARBs/ACE-Is with a shot-to-middle half-life  + CCBs + Diuretics | 6 | 16 |
| ARBs/ACE-Is with a shot-to-middle half-life  + diuretics | 8 | 3 |
| ARBs/ACE-Is with a long half-life   + Beta blockers | 0 | 0 |
| ARBs/ACE-Is with a long half-life   + Beta blockers + CCBs | 2 | 0 |
| ARBs/ACE-Is with a long half-life   + Beta blockers + CCBs + Diuretics | 1 | 0 |
| ARBs/ACE-Is with a long half-life  + CCBs | 16 | 5 |
| ARBs/ACE-Is with a long half-life  + CCBs + Diuretics | 2 | 4 |
| ARBs/ACE-Is with a long half-life  + Diuretics | 3 | 0 |
| Beta blockers + CCBs | 7 | 4 |
| Beta blockers + CCBs + Diuretics | 0 | 0 |
| Beta blockers + Diuretics | 3 | 0 |
| CCBs + Diuretics | 1 | 5 |

ARB, angiotensin receptor blocker; ACE-I, angiotensin-converting enzyme inhibitor; CCB, calcium channel blocker
