# Supplementary material for: Predictors of hypotension during anesthesia induction in patients with hypertension on medication: a retrospective observational study
Source: BMC Anesthesiol. 2022 Nov 11;22:343. doi: 10.1186/s12871-022-01899-9 (PMC9650866; doi:10.1186/s12871-022-01899-9)
Supplement: Supplementary file 5 — Additional file 5: Supplementary Table 5. Baseline blood pressure data of patients receiving monotherapy and multiple therapies. [file 12871_2022_1899_MOESM5_ESM.docx]

Supplemental Table 5 Baseline blood pressure data of patients receiving monotherapy and multiple therapies

| Items | Monotherapy n=181 | Multiple therapies n=214 |
| --- | --- | --- |
| Baseline SAP (mmHg) | 140 [130–152] | 134 [120–145] |
| Baseline DAP (mmHg) | 85 [74–95] | 76 [68–84] |

Data are presented as medians [25^th^–75^th^ percentile].

SAP, systolic arterial blood pressure; DAP, diastolic arterial blood pressure
